# Ultra-compact dual-channel integrated CO_2_ infrared gas sensor

**DOI:** 10.1038/s41378-024-00782-6

**Published:** 2024-10-21

**Authors:** Liyang Feng, Yanxiang Liu, Yi Wang, Hong Zhou, Zhongming Lu, Tie Li

**Affiliations:** 1 State Key Laboratory of Transducer Technology, Shanghai Institute of Microsystem and Information Technology, Chinese Academy of Sciences, Shanghai, 200050 China; 2 University of Chinese Academy of Sciences (UCAS), Beijing, 100049 China

**Keywords:** Electrical and electronic engineering, Optical sensors

## Abstract

Expiratory CO_2_ concentrations can directly reflect human physiological conditions, and their detection is highly important in the treatment and rehabilitation of critically ill patients. Existing respiratory gas analyzers suffer from large sizes and high power consumption due to the limitations of the internal CO_2_ sensors, which prevent them from being wearable to track active people. The internal and external interference and sensitivity limitations must be overcome to realize wearable respiratory monitoring applications for CO_2_ sensors. In this work, an ultra-compact CO_2_ sensor was developed by integrating a microelectromechanical system emitter and thermopile detectors with an optical gas chamber; the power consumption of the light source and ambient temperature of the thermally sensitive devices were reduced by heat transfer control; the time to reach stabilization of the sensor was shortened; the humidity resistance of the sensor was improved by a dual-channel design; the light loss of the sensor was compensated by improving the optical coupling efficiency, which was combined with the amplitude trimming network to equivalently improve the sensitivity of the sensor. The minimum size of the developed sensor was 12 mm × 6 mm × 4 mm, and the reading error was <4% of the reading from −20 °C to 50 °C. The minimum power consumption of the sensor was ~33 mW, and the response time and recovery time were 10 s (@1 Hz), and the sensor had good humidity resistance, stability, and repeatability. These results indicate that the CO_2_ sensor developed using this strategy has great potential for wearable respiratory monitoring applications.

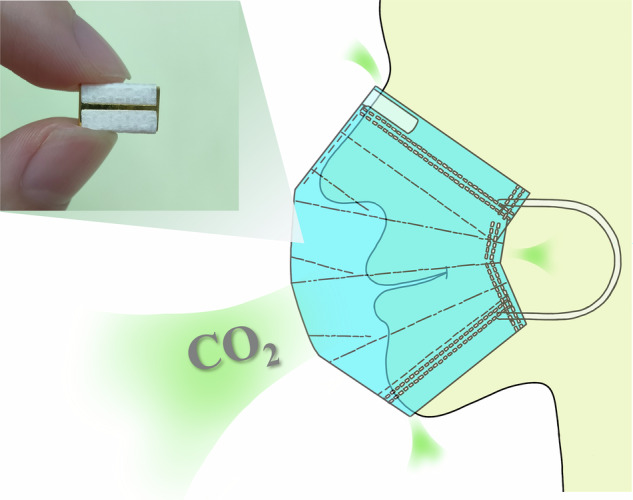

## Introduction

As a major product of the respiratory circulatory system, changes in CO_2_ concentration can directly reflect the physiological condition and metabolic level of a person^1–3^. The respiratory CO_2_ concentration is commonly used in the respiratory management of critically ill patients and is one of the six basic vital signs, along with body temperature, respiration, pulse, blood pressure, and arterial oxygen saturation^4,5^. With the development of sensors in the direction of miniaturization and integration, various traditional vital signs have been realized for portable monitoring^6–11^, which plays an important role in the prevention, diagnosis, treatment, and rehabilitation of today’s diseases^12^. However, in the field of respiratory CO_2_ concentration monitoring, wearable respiratory analyzers have rarely been developed and used due to limitations of the CO_2_ sensor size and power consumption. Thus, they cannot be used for patients suffering from diseases such as acute respiratory distress syndrome, chronic obstructive pulmonary disease, hyperventilation syndrome, and hypoxemia, so their mobility cannot be increased during prevention, treatment, and rehabilitation^13–15^. Cells produce energy, water, and CO_2_ through respiration and ultimately expel CO_2_ from the body through the respiratory system^16^. In sports and health, combining the exhaled CO_2_ concentration with the heart rate and body weight can help athletes, fitness enthusiasts, and obese individuals better calculate energy expenditure^17^, so wearability is necessary to meet the needs in this field.

Currently, there are various sensors to monitor the CO_2_ gas concentration and non-dispersive infrared (NDIR) technology has become the preferred method in respiratory monitoring because of its advantages of good stability and selectivity, high precision and sensitivity, wide measurement range, and long lifetime^18–20^. The size of commercially available CO_2_ sensors is commonly ~5 cm^3^, and people do not consider putting a CO_2_ sensor of this size in front of the face to realize wearable respiratory monitoring. Breaking the size barrier of NDIR CO_2_ sensors is a key aspect in realizing wearable respiratory monitoring. Hodgkinson et al. developed a compact NDIR CO_2_ gas sensor using a discrete component assembly method^21^. The package size of the sensor is 20 mm in diameter × 16.5 mm in height, the internal light source and detectors are placed back-to-back, and an optical path length of ~32 mm and an optical coupling efficiency of ~9.4% from the light source to the two detectors are achieved by designing multiple reflective surfaces. The bulk of the sensor’s volume is from the detectors, which are packaged in a 9-mm-diameter TO-5 can. Tan et al. integrated a microbulb infrared light source and four encapsulated pyroelectric detectors in a metal optical chamber with dimensions of 20 mm diameter × 10 mm height to simultaneously detect CO_2_, CO, and CH_4_ gas concentrations^22^. The sensor has an optical path length of 26 mm through two reflections of infrared light, and the accuracy of the CO_2_ measurements is (±0.05% Vol) in the range of 0 to 48,000 ppm range. Chowdhury et al. used an infrared device in a surface-mounted device to reduce the size of the sensing area of the CO_2_ sensor to 16 mm × 10 mm × 10 mm, which can be used for respiratory CO_2_ detection after being connected to an external air tube; the introduction of MEMS infrared effectively reduces the power consumption of the sensor and improves the response frequency^23^. CO_2_ sensors assembled with discrete components can only attempt to optimize the layout of the light source and detector, and much effort has been spent on reducing the size of the optical gas chamber. In addition, the dual detectors in TO-5 are not conducive to the efficient collection of incident infrared light and generate an ~40% loss in optical coupling efficiency around the detectors.

Yang et al. fabricated a dual-optical path silicon-based integrated optical gas chamber with dimensions of 10 mm × 10 mm × 1 mm via the MEMS process and achieved an optical coupling efficiency of ~17.6% from the MEMS infrared source to the pyroelectric detector through simulation^24^. Zhang et al. improved the total optical coupling efficiency to 29% and realized a 19.75-mm optical range length based on Yang et al. and used a thermally isolated design to reduce the thermal interference of the light source on the detector^25^. Jia et al. fabricated an optical gas chamber with a package size of 6 mm × 6 mm based on waveguide technology, realized a 34-mm optical path length through multiple reflections, and used a bare light emitting diode (LED) and photodiodes to fabricate an on-chip integrated CO_2_ sensor. In this setup, the coupling efficiency between light source and input waveguide was only 8%, and that between output waveguide and photodiode was only 30%^26,27^. Although optical gas chambers fabricated using silicon-based integration technology can significantly reduce the size and cost of the sensors, the lower light utilization makes the sensors less accurate. To realize this application, this type of sensor must be improved by increasing the optical power of the light source or optical coupling efficiency of the optical gas chamber to increase the accuracy of the sensor.

Eberl et al. developed an integrated CO_2_ sensor using the photoacoustic principle by integrating a MEMS emitter and a MEMS microphone inside a metal chamber of ~10 mm × 10 mm × 10 mm, which can operate in the range of −40–90 °C with an accuracy of 10% (100–40,000 ppm) and a response time of 20 s^28^. Scholz et al. designed a 12-mm × 8-mm × 4-mm photoacoustic CO_2_ sensor using an LED and a microphone as the core components to detect CO_2_ concentrations in the range of 500–5000 ppm^29^. The sensor was constructed by embossing an ellipsoidal reflective surface on polymethylmethacrylate (PMMA) via thermocompression to create an optical gas chamber with an optical path length of 12 mm, and the typical power consumption of the sensor was 48.2 mW. CO_2_ sensors based on the photoacoustic principle have been successfully commercialized owing to their small size, but the low CO_2_ measurement frequency of this type of sensor directly leads to a slower response time, and the typical response time of this type of commercially available CO_2_ sensor is 60 s (t63)^30^.

**Fig. 1:**
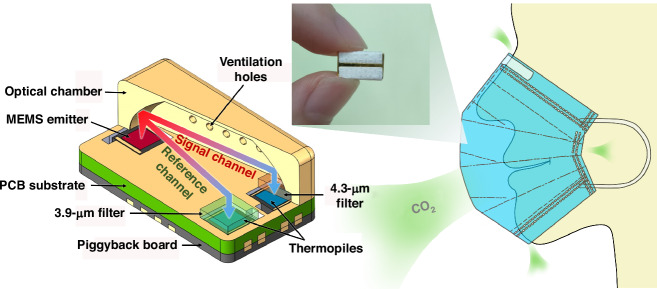
Ultra-compact dual-channel integrated CO_2_ infrared gas sensor schematic and wearable breath detection device

In summary, there are many methods to unilaterally reduce the size of a sensor, but other properties must be sacrificed in exchange. Two main obstacles prevent CO_2_ sensors from achieving small size and good performance: the high temperature generated by the light source in a small package reduces the output signal‒to‒noise ratio of thermal detectors, and the sensitivity of NDIR gas sensors is positively correlated with the optical path length (size of the optical gas chamber). In our previous work, we developed an ultra-compact single-channel CO_2_ infrared gas sensor by high-density integrated packaging of a high-emissivity MEMS emitter, a high-detectivity thermopile detector, and a high-coupling-efficiency injection-molded optical gas chamber, which achieved compatibility between small size and good performance^31^. In addition to their small size, low power consumption, good accuracy, and fast response, respiratory CO_2_ sensors should also satisfy the requirements of practical application scenarios. On the one hand, because the relative humidity of human exhaled breath is close to 100% and the temperature is ~36 °C^19,32^, the CO_2_ sensor for human exhaled CO_2_ concentration monitoring must operate below 40 °C with good humidity resistance. On the other hand, because respiratory monitoring equipment needs to work quickly and provide accurate comparative data results in emergency scenarios, the stabilization time and long-term stability of the sensor become extremely important. In this work, we developed an ultra-compact dual-channel integrated CO_2_ infrared gas sensor following the high-density integrated packaging method (Fig. 1), designed a dual-optical path reflective-concentration structure with an optical coupling efficiency of 78% via ray tracing simulation, and used the differential method in the dual-channel architecture. This method filtered out the additional reading error generated by the infrared light absorption by water molecules in the respiratory gas and corrected the reading drift caused by changes in internal temperature and emission efficiency of the sensor. In addition, by introducing an amplitude trimming network in the signal processing circuit, the sensitivity reduction due to the shortening of the optical path length is equivalently compensated. The ultra-compact dual-channel integrated CO_2_ infrared gas sensor based on this strategy compensates for the shortcomings of the current NDIR CO_2_ gas sensors, so it simultaneously has a small size, high stability, good accuracy, low power consumption, low cost, and fast response time. Thus, this sensor has a wide range of application prospects in wearable respiratory monitoring.

## Materials and methods

### Structural design

**Fig. 2:**
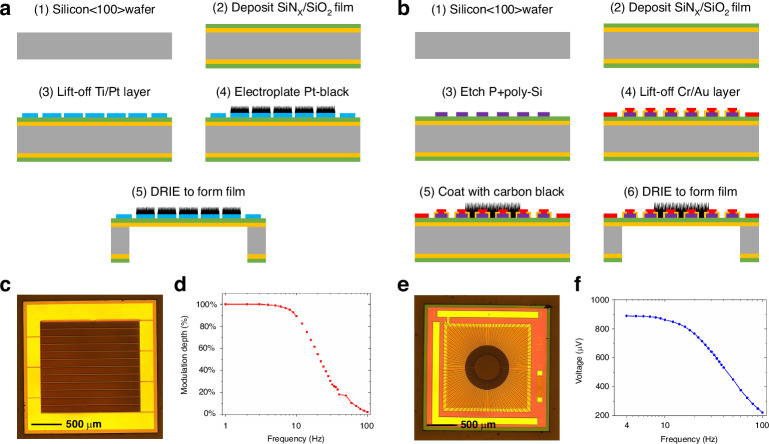
MEMS emitter and thermopile and their process flow diagrams and test results. **a** Process flow diagram and picture of the MEMS emitter. **b** Process flow diagram and photograph of the thermopile. **c** MEMS emitter microscope image. **d** MEMS emitter modulation depth test results. **e** Thermopile microscope image. **f** Thermopile response time constant test results

Typically, NDIR gas sensors quantify the gas concentration as an electrical signal through three components: an infrared light source, an infrared detector, and an optical gas chamber^33^. Proper selection of these components can enhance the sensor’s advantages in terms of power consumption, cost, and size. Conventional infrared light sources mostly use tungsten bulbs, which provide high optical power through high power consumption and can provide strong infrared signals to detectors, but they are usually large in size. Moreover, LEDs are small in size and low in power consumption, and they can directly emit narrowband infrared light by omitting the narrowband filter, which is a traditional component. However, they are usually expensive^34^ and emit a narrowband spectrum with a full width at half maximum (FWHM) five times greater than the FWHM of narrowband filters, which weakens the selectivity of the sensor. Therefore, we adopt an MEMS emitter as the infrared light source of the sensor, which has a small size, appropriate power consumption, stable performance, a low cost, and high luminescence efficiency in the mid-infrared wavelength band. Figure 2a shows the fabrication process of the high-emissivity MEMS emitter, which mainly includes the following steps:

1. The substrate material is a 4-inch n-type single-crystal silicon wafer with a crystal orientation <100> and a thickness of 400 µm.
2. SiN_X_/SiO_2_ low-stress composite films with a thickness of 2 µm were prepared as support films for the heating resistors via thermal oxidation and low-pressure chemical vapor deposition (LPCVD) processes.
3. A 400-nm Pt heating resistor and a 40-nm Ti adhesion layer were prepared via lift-off.
4. A 300-nm Pt-black layer was electroplated.
5. A deep reactive ion etching (DRIE) process was used to etch the back side of the graphic area to release the suspended film structure.

Figure 2c shows a light source fabricated via the MEMS process. The modulation depth of this light source was tested by pairing it with a thermopile infrared detector. The modulation depth is defined as the percentage of the detector’s output response after the switching frequency of the light source has been modulated to the detector’s output response when the light source is operated at a low frequency^35^. Here, the detector output response when the light source operates at 1 Hz was selected as the reference value for 100% modulation depth. Figure 2d shows the curve of the MEMS emitter modulation depth versus frequency, where the MEMS emitter has a modulation depth greater than 99% within 5 Hz with an excellent temperature shift speed.

For infrared detectors, the three main choices are photodiodes, pyroelectric detectors, and thermopile detectors. Photodiodes are small in size, are often used in conjunction with LEDs, and have the disadvantages of LEDs. Pyroelectric detectors have less thermal noise than thermopile detectors, but thermopile detectors are easier to use because they are smaller and more cost-effective. Figure 2b shows the fabrication process of the high-detectivity thermopile detector, which consists of the following steps:

1. The substrate material is a 4-inch n-type single-crystal silicon wafer with a crystal orientation <100> and a thickness of 400 µm.
2. SiN_X_/SiO_2_ low-stress composite films with a thickness of 1.35 µm were prepared as support films for thermocouples via thermal oxidation and LPCVD.
3. The LPCVD process was used to prepare 800-nm low-stress polysilicon and ion-implant boron with an energy of 90 Kev and a dose of 9 × 10^15^ cm^−2^, which was etched to form P+poly-Si thermoelectric arms and oxidized a layer of SiO_2_ as an insulating layer on its surface.
4. The 300-nm Au thermoelectric arms and 30-nm Cr adhesion layer were prepared via lift-off.
5. A 200-nm carbon black layer was coated in the center of the graphic area via spraying.
6. A DRIE process was used to etch the back side of the graphic area to release the suspended film structure.

There were 88 pairs of thermocouples on the surface of the film area of the thermopile infrared detector (Fig. 2e). The performance parameters of the thermopile were determined through a blackbody furnace infrared radiation system. The infrared radiation temperature of the blackbody furnace is 227 °C, and the power density radiated to the surface of the detector is 9 W m^2^ ^36^. Figure 2f shows the variation curve of the output response of the thermopile detector concerning the frequency, which has a high detection rate and a small response time constant. Table 1 shows the performance parameters of the thermopile.

**Table 1:** Performance parameters of the thermopiles

Absorber area (mm^2^)	Electrical resistance (KΩ)	Output voltage (μV)	Responsivity (V W^−1^)	Detectivity (10^7^ cm Hz^1/2^ W^−1^)	Response time constant (ms)
1.44	108	887	68.45	1.97	5.13

For the optical gas chamber, the injection molding method helps optimize the design of the internal optical reflective surfaces to improve the optical coupling efficiency, and the mass production method has cost advantages. The dimensions of the optical gas chamber are determined after the optical path length has been determined, which affects the performance of the sensor and usually are designed according to Beer‒Lambert law^37^:

1 $$I={I}_{0}{{\rm{e}}}^{-{kcl}},$$

where *I*_0_ is the light intensity of the light source, *I*_1_ is the light intensity detected by the detector, *k* is the absorption coefficient, *c* is the gas concentration, and *l* is the optical path length. The response signal *U*_1_ output by the detector is proportional to the detected light intensity *I*_1_. Taking the response signal *U*_0_ detected by the detector when the carbon dioxide concentration is 0% as a reference, the normalized absorption rate (NAR) is obtained:

2 $${NAR}=\frac{{U}_{0}-{U}_{1}}{{U}_{0}}=\frac{{I}_{0}-{I}_{1}}{{I}_{0}},$$

Substituting Eq. (1) into Eq. (2) yields:

3 $${NAR}=1-{{\rm{e}}}^{-{kcl}},$$

Equation (3) shows that the optical path length is negatively correlated with the range of the sensor for a given model. For the sensor to have the proper range to detect changes in respiratory CO_2_ concentration, the appropriate optical path length should be selected to guide the design of the optical gas chamber. Vincent et al. reported an infrared CO_2_ gas sensor for respiratory CO_2_ detection using a packaged SOI light source and a thermopile detector as the core element and compared the response of the thermopile detector for three optical path lengths: 10 mm, 20 mm, and 40 mm^19^. When the CO_2_ concentration was varied from 0 to 50,000 ppm, the 40-mm sample had a similar response to the 20-mm sample, and the response of the 10-mm sample was only 14% smaller than that of the 20-mm sample. Akram et al. used PMMA/Au to fabricate a low-cost miniature optical gas chamber and experimentally demonstrated that absorption saturation occurs at CO_2_ concentrations of 40,000 ppm and 80,000 ppm for 50 mm and 20 mm optical channels, respectively^38^. Therefore, for the sensor to detect CO_2_ in the range of 400–100,000 ppm, the optical path length of the sensor should be 10–15 mm. Because certain reflections in the optical gas chamber increase the optical path length, we fabricated an optical gas chamber with dimensions of 12 mm × 6 mm × 2 mm.

Since the sensor should detect CO_2_ changes in respiratory gas, it must resist humidity to function in high-humidity environments, where the relative humidity (RH) in human exhaled breath is close to saturation^32^. In addition, during the continuous operation of the sensor, the MEMS emitter as a high-temperature device will lose its emission efficiency with increasing operating time, and the electronic components distributed around the MEMS emitter will suffer from the temperature drift, so the high-temperature characteristics directly affect the stabilization time and long-term stability of the sensor. Here, we adopted a dual-channel structure to solve these problems, used two thermopile detectors to obtain responses of the 4.26-μm (signal-channel) and 3.9-μm (reference channel) wavelength infrared light, and normalized the responses of the signal-channel and reference channel in the post-processing circuit to obtain relatively stable readings.

Figure 1 shows the structure of the ultra-compact CO_2_ sensor, where the MEMS emitter and the two thermopile detectors are in the same plane, and they are placed in insulated slots on both sides of the device carrier board. The optical gas chamber is covered on top of the device carrier board to provide the gas-absorbing environment and to conduct infrared light from the MEMS emitter to the two thermopile detectors. Finally, a post-processing circuit board is added under the device carrier board to realize signal processing and conversion.

### Simulation

#### Thermal

**Fig. 3:**
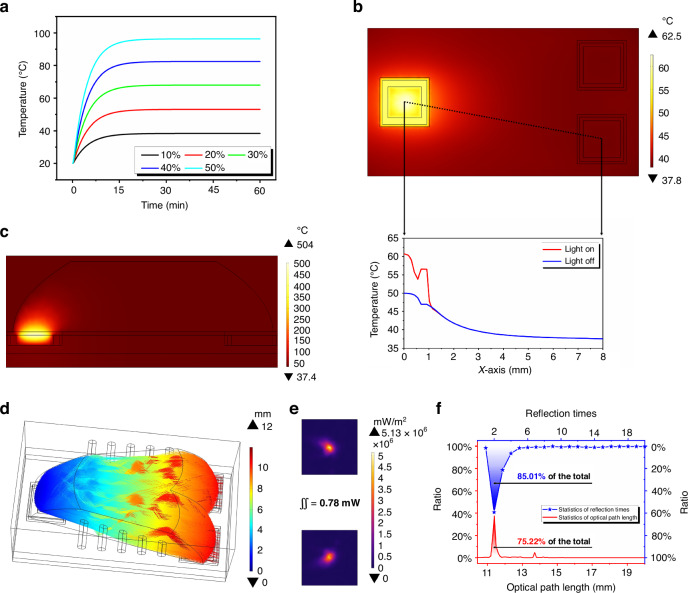
Sensor simulation results. **a** Temperature variation curves around the thermopile in different duty cycles. **b** Temperature distribution of the bottom cross-section of the MEMS emitter and thermopile when the MEMS emitter is operating in the 10% duty cycle. **c** Temperature distribution of the longitudinal section of the sensor when the MEMS emitter is operating in the 10% duty cycle. **d** Ray tracing simulation results. **e** Irradiance of the thermopile surface via ray tracing. **f** Statistics of the number of ray reflections and optical path length

Inside an ultra-compact integrated CO_2_ infrared gas sensor, a MEMS emitter operates by generating high temperatures to radiate mid-infrared light. However, thermopile detectors, which output their response through a temperature difference, are typically very sensitive to temperature. Therefore, the size reduction poses a challenge for thermal management. Here, we simulated the temperature equilibrium process and transfer trend inside the sensor using the transient simulation method in COMSOL software. In the simulation, the light source was used as the main heat source, and air heat transfer was considered the main heat dissipation method. Figure 3a shows the temperature equilibrium process around the detector at different turn-on duty cycles within one hour after the sensor was powered on. When the light source operates at ~500 °C, the temperature around the detector decreases by ~60% in the 10% duty cycle operation mode compared with the conventional 50% duty cycle operation mode. Figure 3c shows the longitudinal temperature profile of the sensor after one hour of operation. The maximum temperature of the membrane region of the MEMS emitter is 504 °C, and it can efficiently radiate infrared light of ~4.26 µm according to Wien’s displacement law^39^. Figure 3b shows the temperature distribution of the bottom surface of the MEMS emitter and thermopiles when the light is on. The temperature change curve from the MEMS emitter to the thermopile shows that the surface of the PCB corresponding to the center of the MEMS emitter has the highest temperature, which is ~62.5 °C when the light is on and 50 °C when the light is off, and the temperature rapidly decreases to ~37.8 °C along the dotted line because of isolation grooves between the MEMS emitter and the surrounding cavity. In the real environment, the actual temperature near the thermopiles and on the surface of the sensor is lower than the simulation results, considering the multi-layer structure inside the sensor to assist in thermal insulation and convective heat transfer outside. The thermal simulation results show that a shorter duty cycle reduces the power consumption and minimizes the influence of the MEMS emitter on the thermopiles if the response time requirements of the light source and detector are satisfied.

#### Optical

Efficient utilization of infrared optical signals from MEMS emitters can overcome noise limitations and enhance the sensor sensitivity. Therefore, the compound parabolic concentrator (CPC) design method^40^ was referenced during the design of the optical gas chamber and applied to the reflective surfaces at the top of the MEMS emitter and thermopile detectors. The use of the CPC enables the light to be transmitted in an approximate point-to-point fashion, so the thermopiles have hotter thermal junctions and obtain a strong response signal. Ray tracing simulation was performed using the COMSOL software; the settings were: area of the emitting region of the MEMS emitter: 0.6 mm in diameter; divergence angle: 90°; total optical power of the outgoing beams: 1 mW with 10,248 strips. Figure 3d shows the ray tracing simulation results of the CO_2_ infrared gas sensor fabricated in this study, where the blue color indicates the beginning of the infrared light ray, and the red color indicates the end of the infrared light ray. The simulation results show that the NDIR transmission model can transmit most of the emitted infrared light rays from the light source to one end of the infrared detectors. Since the rays do not involve the sides, air holes of the optical chamber were set on both sides to increase light utilization. The rays in Fig. 3d are statistically analyzed in Fig. 3f. In this simulation model, 85.01% of the rays reached the surface of the film area of the thermopile detector after 2–4 reflections, of which ~40% had to pass through only two reflections, and rays with optical path lengths of 11–12 mm accounted for 75.22%. Figure 3e shows the infrared light capture results on the surface of the membrane area of the thermopile detectors, and the maximum irradiance on the surface of the membrane area of the thermopile detectors can reach 5.13 × 10^6^ mW/m^2^. By integrating the irradiance on the surface of the membrane area of the thermopile detectors, we obtained that the two thermopile detectors collected 0.78 mW of optical power, which implies that the maximal optical coupling efficiency of this optical gas chamber is 78%.

### Signal processing

A shorter optical path length improves the range of the sensor but reduces the sensitivity of the sensor. Here, we indirectly enhanced the sensitivity of the sensor by extracting and amplifying the amount of signal change in the post-processing circuit. First, we must establish the relationship between the boosting multiplier *α* of the signal change amount acquired by the analog-to-digital converter (ADC) module and the sensitivity *S*. When the signal change amount is not extracted, the absorbed light intensity ${I}_{{before}}$ in the optical chamber at this time can be introduced from Eq. (1):

4 $${I}_{{before}}={I}_{0}-{I}_{1}={I}_{0}(1-{{\rm{e}}}^{-{kcl}}),$$

Combined with formula (3), the signal change amount collected by the ADC module at this time should be ${U}_{{before}}$. After the signal has been extracted and amplified in the signal processing process, the signal change amount collected by the ADC module can be improved by $\alpha$ times; at this time, ${U}_{{before}}$ becomes ${U}_{{after}}$:

5 $$\alpha =\frac{{U}_{{after}}}{{U}_{{before}}}=\frac{{I}_{{after}}}{{I}_{{before}}},$$

At this point, the relationship between absorbed light intensity ${I}_{{after}}$ and CO_2_ concentration c in the optical gas chamber can be expressed as:

6 $${I}_{{after}}=\alpha {I}_{0}(1-{{\rm{e}}}^{-{kcl}}),$$

Let $\beta ={U}_{0}/{I}_{0}$, where *β* is constant in the NDIR model, which is directly proportional to the optical coupling efficiency; thus, combining Eq. (2) and Eq. (6) yields the equation of the sensor sensitivity at this time:

7 $$S=\frac{{\rm{d}}{U}_{a}}{{\rm{d}}c}=\alpha \beta {kl}{{\rm{e}}}^{-{kcl}},$$

where *S* is the sensitivity of the sensor, *α* is the increase in signal variation acquired by the ADC, *β* is the optical-electrical conversion efficiency of the infrared detector, *k* is the absorption coefficient, *c* is the gas concentration, and *l* is the optical path length. Equation (7) shows that when the sensitivity of the sensor cannot be enhanced by increasing the optical path length, in addition to enhancing *β* by improving the optical coupling efficiency, $\alpha$ can be enhanced by extracting and amplifying the amount of signal changes captured by the ADC module, which ultimately enhances the sensor sensitivity.

In a typical processing circuit, when the output signal of the thermopile detector has a peak-to-peak value of 1 mV, an operational amplifier and a filtering network are used to amplify the signal to ~2 V, which is subsequently captured by the ADC module, and the amount of signal change is calculated. However, under the specified sensor range and optical path length, the infrared light emitted by the MEMS emitter is not fully absorbed by the gas to be measured in the optical gas chamber. Thus, the output signal of the thermopile detector has the peak-to-peak voltage change and is superimposed with an in-phase and same-frequency carrier component, which is amplified with the peak-to-peak voltage change amount and occupies the ADC sampling bits. We can crop out this carrier component to extract the part of the signal that contains the change. After the operational amplifier re-amplification, the ADC module can capture more peak-to-peak voltage changes.

**Fig. 4:**
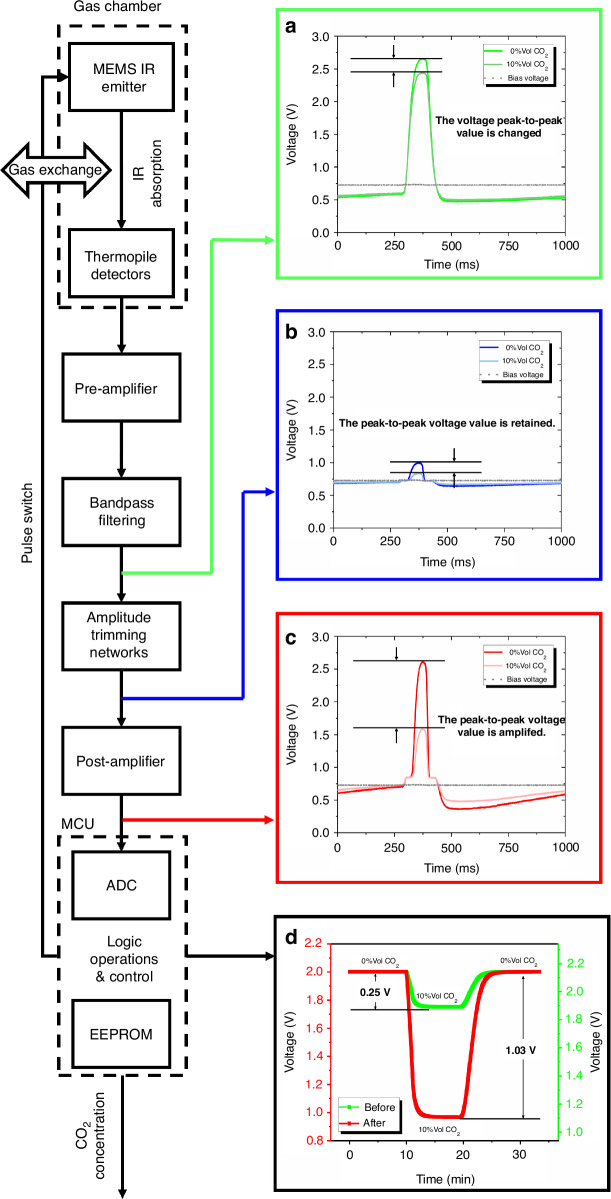
Signal processing flowchart. **a** Typical circuit output waveform. **b** Trimmed signal waveform. **c** Amplified trimmed signal waveform. **d** Concentration-response results before and after amplitude trimming

Figure 4 shows the signal processing process of the ultra-compact CO_2_ infrared gas sensor. The microcontroller unit (MCU) provides the MEMS emitter with a pulse signal with a frequency of 1 Hz and a duty cycle of 10%, and the thermopile detector generates the corresponding signal, which is captured by the ADC chip after pre-amplification, bandpass filtering, amplitude trimming, and post-amplification. Finally, the electrical signal is converted into a concentration reading in the MCU. Here, we present the output waveforms of the sensor at 0% Vol and 10% Vol CO_2_ concentrations and compare the changes in waveforms before and after signal extraction. Figure 4a shows the waveform of the signal with the carrier component, which is normally acquired by the ADC module, with a small amount of peak-to-peak voltage change (the main change is from the wave crest). Figure 4b shows the waveform after the signal passes through the amplitude trimming network, which retains the voltage change generated by gas absorption and removes the carrier component. Next, we amplified the waveform in Fig. 4b again to obtain the final signal waveform in Fig. 4c, which reflects the absorption of infrared light by the gas molecules and enables the ADC module to capture a large peak-to-peak voltage change. Finally, Fig. 4d shows the response curve of the sensor to the gas concentration, and the sensitivity of the sensor is improved by more than 4 times.

## Results and discussion

### Characteristic test

**Fig. 5:**
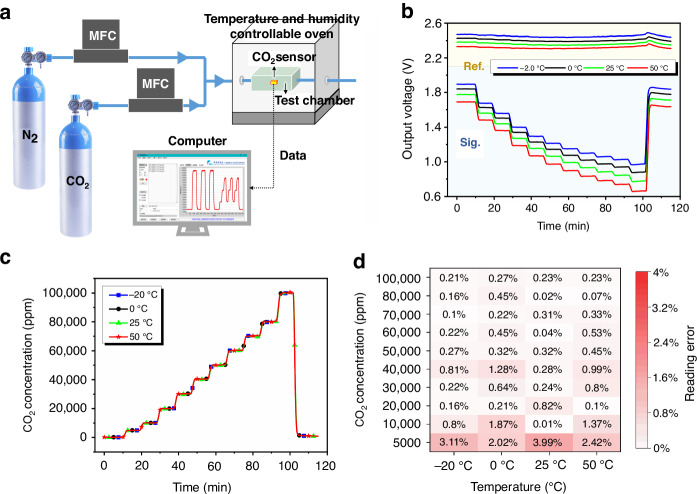
Calibration and testing experiments of the ultra-compact CO_2_ sensor. **a** Calibration and testing platform for the ultra-compact CO_2_ sensor. **b** Original output voltage values of the ultra-compact CO_2_ sensor at different temperatures and CO_2_ concentrations. **c** Accuracy test results of the ultra-compact CO_2_ sensor at different temperatures and CO_2_ concentrations. **d** Reading errors of the ultra-compact CO_2_ sensor at different temperatures and CO_2_ concentrations

The ultra-compact dual-channel integrated CO_2_ sensor has a minimum size of 12 mm × 6 mm × 4 mm. The sensor is powered by a minimum of 3 V, has an on-light current of 72 mA and an off-light current of 4 mA, and consumes 33 mW of power at a 1-Hz operating frequency with a duty cycle of 10%. Figure 5a shows the calibration and testing platform of the ultra-compact CO_2_ sensor. In this platform, the carrier gas is nitrogen with a concentration of 100% Vol, and the gas to be tested is carbon dioxide with a concentration of 10% Vol. The two gas paths are connected to the gas mass flow controller (MFC) to control the gas flow rate; then, the two gases are mixed through the three-way valve and sent into the test chamber that contains the CO_2_ sensor inside the temperature- and humidity-controllable oven (THCO). Finally, the CO_2_ sensor sends the real-time test results to the host computer software through the serial port for display and storage. Before testing the reading error of the ultra-compact CO_2_ sensor for different CO_2_ gas concentrations, we performed a calibration test on the sensor. The calibration process helps us obtain the output voltage values of the sensor at different temperatures and CO_2_ concentrations (Fig. 5b), which were fitted to a curve and written into the internal memory chip of the sensor. In subsequent tests, the sensor converted the real-time voltage signal into CO_2_ concentration values based on the calibration results.

After calibration, the accuracy characteristics of the ultra-compact CO_2_ sensor were tested. During the test, the temperature of the test chamber was controlled by the THCO at four gradients of −20 °C, 0 °C, 25 °C and 50 °C, and the humidity of the test chamber was 0% RH. The total flow rate of the gases in the pipeline after mixing was set to a constant 400 sccm; then, the flow rate ratios of the two gases were controlled by an MFC to obtain CO_2_ concentrations of 0% Vol, 0.5% Vol, 1% Vol, 2% Vol, 3% Vol, 4% Vol, 5% Vol, 6% Vol, 7% Vol, 8% Vol, and 10% Vol. Figure 5c shows the results of the accuracy test, where the sensor has a clear indication step for different CO_2_ concentrations, and the curves are highly coincident at different temperatures. The results in Fig. 5c were organized to obtain Fig. 5d, which shows the reading error of the sensor at each concentration point at different temperatures. The maximum reading error of the sensor is less than 4% of the standard value in the CO_2_ concentration interval of 0–10,000 ppm and less than 2% of the standard value in the CO_2_ concentration interval of 10,000–100,000 ppm.

### Response speed test

**Fig. 6:**
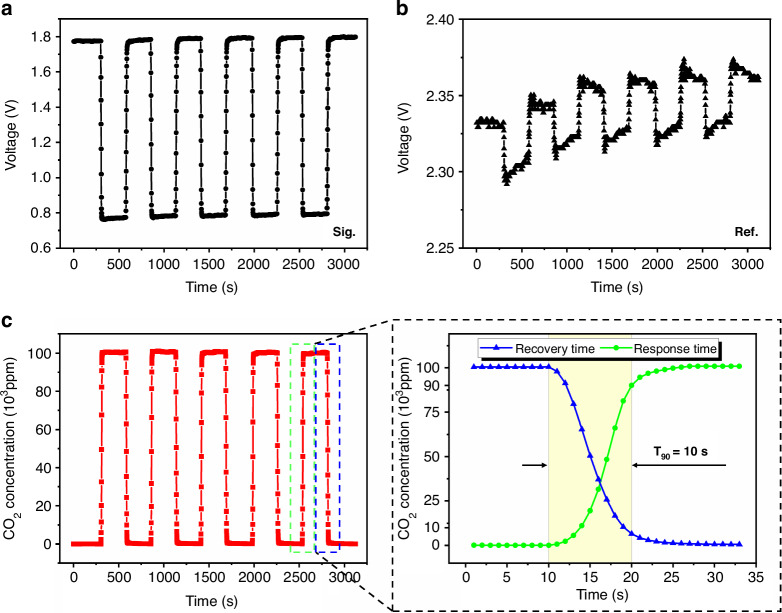
Response time and recovery time test results. **a** Actual voltage value output by the signal-channel. **b** Actual voltage value output from the reference channel. **c** Result of the calculated sensor reading

The response speed of the ultra-compact CO_2_ sensor was tested by alternately passing pure N_2_ or 10% Vol CO_2_ gas through a valve controlled at 5-mins intervals. The reading frequency of the ultra-compact CO_2_ sensor was set to 1 Hz, and the time to change the sensor’s reading from the beginning value to 90% of the standard concentration value was its response time; similarly, the time to change the sensor’s reading from the beginning to 10% of the standard concentration value was its recovery time. Figure 6 shows the response time and recovery time of the ultra-compact CO_2_ sensor. When gases are switched, the output voltage values of the sensor signal-channel (Fig. 6a) and the reference channel (Fig. 6b) are rapidly transformed. Fitting the results of Fig. 6a with those of Fig. 6b yields the results in Fig. 6c, which indicate that the response time and recovery time of the sensor are both 10 s at an operating frequency of 1 Hz.

### Humidity test

The humidity characteristics of the ultra-compact CO_2_ sensor are very important for monitoring respiratory gas. The temperature of the gas exhaled by a normal person from the mouth is ~36 °C, and the relative humidity of the gas is close to saturation. The humidity characterization test platform is similar to that in Fig. 5a with the following differences: the gas in both cylinders was CO_2_ with a concentration of 4% Vol; a bubbler device was added after one of the MFCs to provide humidity, and the bubbler was placed in the THCO; a humidity sensor was added to the test chamber to record the humidity changes in the test environment. During the test, the concentration of CO_2_ gas in the test environment was controlled to be 4% Vol, and the flow rate of the two gases was proportional to the MFC to produce different humidity levels. The test time of each humidity point was 1 h, and the ambient temperature was controlled by the THCO, which was 20 °C, 30 °C, 40 °C, and 50 °C.

**Fig. 7:**
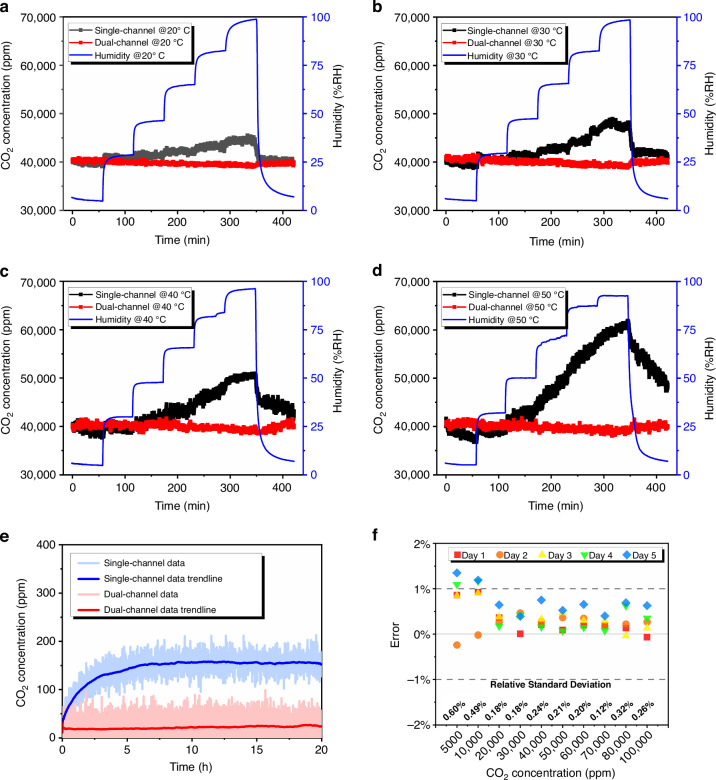
Test results of the ultra-compact CO_2_ sensor. **a**–**d** Results of variable-humidity tests of the sensor at 20 °C, 30 °C, 40 °C, and 50 °C. **e** Stability test results of the sensor using the single-channel direct output and dual-channel differential output. **f** Sensor reading error test results and repeatability error test results at 25 °C

Figure 7a–d shows the humidity characteristics of the ultra-compact CO_2_ sensor at four temperature points, where we present both single-channel fitted data and two-channel differential data at each temperature point. The test results show that without the reference channel to correct for water vapor interference, the reading error of the sensor at the same temperature increases with humidity, and a higher ambient temperature at the sensor with identical humidity corresponds to more pronounced water vapor interference. After the introduction of the reference channel, the sensor was unaffected by humidity changes within 50 °C and had stable reading results, which can satisfy practical application requirements.

### Stability and repeatability tests

Stability testing of the ultra-compact CO_2_ sensor was accelerated at 50 °C under pure nitrogen. After the temperature and gas environment stabilized, the sensor was powered on and immediately zero-calibrated; then, the sensor readings were continuously observed and recorded using the host computer software. Normally, after the CO_2_ sensor has been powered on and begins working, the overall temperature of the sensor increases with the periodic switching of the light source and eventually reaches a steady state. The temperature of the cold junction of the thermopile also reaches equilibrium during the periodic heat absorption and exothermic processes; then, the sensor can output a stable signal. Figure 7e shows the stability test results of the ultra-compact CO_2_ sensor, which reveals the advantage of the dual-channel structure over the single-channel structure in resisting temperature drift. After the sensor had been powered on for a period of time, the readings using single-channel data drifted over 10 h and eventually stabilized as the internal state of the sensor stabilized, whereas the readings using dual-channel data filtered out this temperature change process through differential operations, which enabled the sensor to immediately output stable readings.

The experimental platform for the repeatability testing of the ultra-compact CO_2_ sensor was identical to that in Fig. 5a, and the THCO continuously maintained the internal temperature at 25 °C throughout the experimental period. The concentration gradient test in Fig. 5c was performed every day after the sensor was powered on and operated; then, the reading errors of the sensor at each standard concentration point were counted and plotted in Fig. 7f. Finally, the test data of the sensor for five days were sorted and substituted into the formula of relative standard deviation to obtain its repeatability error at 10 standard concentration points, which were plotted in Fig. 7f. The test results show that the repeatability error of the sensor does not exceed 0.60% in the CO_2_ concentration interval of 0–10,000 ppm and 0.32% in the CO_2_ concentration interval of 10,000–100,000 ppm.

### Applications

We designed a wearable exhaled CO_2_ monitoring system based on a mask platform to perform a pilot study of the ultra-compact CO_2_ sensor. The ultra-compact CO_2_ sensor was mounted inside the mask, which was tuned to operate at 5 Hz with a powered battery and a Bluetooth transmission module outside. To ensure that the results of the pilot study are comparable, we wore the mask on a head model. Since the CO_2_ concentration in human exhaled breath is ~4% vol, standard CO_2_ gas with a concentration of 4% vol was fed into the mask via a catheter, and the gas was passed through a bubbler and THCO to ensure that the temperature and humidity were correct.

**Fig. 8:**
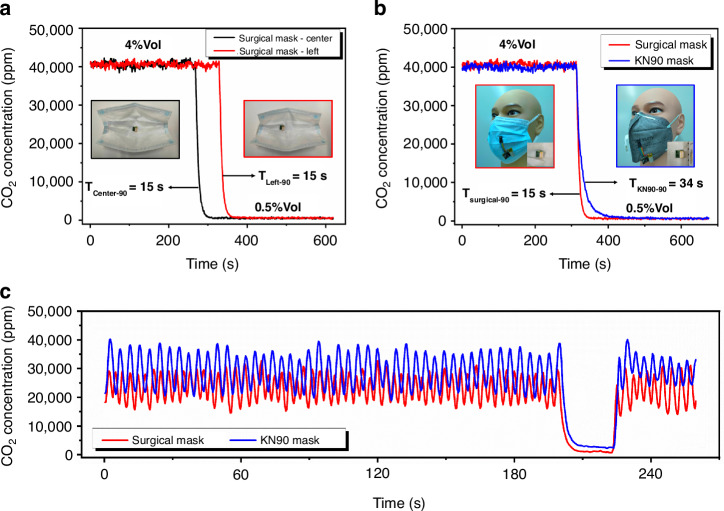
Ultra-compact CO_2_ sensor application experiments. **a** Effects of different placements of the CO_2_ sensor in the mouthpiece on its readings. **b** Difference in CO_2_ sensor readings for different masks. (c) Results of the exhaled breath monitoring experiments when the CO_2_ sensor was in different masks

First, we compared the readings of the ultra-compact CO_2_ sensor when it was mounted in different positions inside the surgical mask. Considering the limited space inside the mask, the CO_2_ sensor could only be installed in the center or the side of the surgical mask during the test. Figure 8a shows that when the ultra-compact CO_2_ sensor was installed in the center of the surgical mask, the reading error at the 4% Vol concentration point was 1.56%; when the ultra-compact CO_2_ sensor was installed on the left side of the surgical mask, the reading error at the 4% Vol concentration point was 1.62%; the CO_2_ leakage time inside the surgical mask was 15 s at all positions, and the sensor placement had almost no effect on the reading results. We subsequently tested the reading status of the ultra-compact CO_2_ sensor when it was installed inside different masks. Figure 8b shows the test results. When the ultra-compact CO_2_ sensor was mounted on one side of the KN90 mask, the reading error at the 4% Vol concentration point was 0.12%, and the CO_2_ gas leakage time inside the mask was 34 s. Finally, an adult healthy male was selected to wear each of the two masks with the ultra-compact CO_2_ sensor, and Fig. 8c shows the test results. The subject sat still for 10 min before the test, and successive tests of normal breathing, stopping breathing, and resuming breathing were performed to obtain CO_2_ readings. The test results of the wearable exhaled CO_2_ monitoring system show that the respiration rate of the surgical mask was 18 breaths/minute, and the variation interval of the CO_2_ concentration in the mask was 1.5–3% Vol; the respiration rate of the KN90 mask was 14 breaths/minute, and the variation interval of the CO_2_ concentration in the mask was 2–4% Vol. A comparison of the test results reveals differences in the test results when the ultra-compact CO_2_ sensor is installed on different mask platforms, and the CO_2_ concentration data in the breath of the subjects should be analyzed according to different mask platforms.

## Conclusions

In summary, an ultra-compact dual-channel integrated CO_2_ infrared gas sensor was introduced. The sensor selects a MEMS emitter with high emissivity, good stability, and low cost as the light source and two thermopile detectors with high-selectivity and high-cost effectiveness as the infrared detectors; then, a low-cost optical gas chamber was fabricated via injection molding. The temperature distribution in the high-density package structure was simulated and analyzed, and the drift problem of the sensor was suppressed by designing a dual-optical path structure on the optical gas chamber. The optical simulation results of the sensor included the optical path length of the sensor and number of reflections, realized an optical coupling efficiency of ~78%, and multiplied the number of signal changes collected by the ADC module by adding an amplitude trimming network in the post-processing circuit. This circuit t can compensate for the sensitivity reduction caused by the decrease in optical path length. The minimum size of the sensor is 12 mm × 6 mm × 4 mm, the minimum power consumption of the sensor is ~33 mW at 3 V supply and 1 Hz operating frequency, and the response time and recovery time of the sensor are 10 s (t90). The reading error of the sensor is less than 4% of the reading in the 0–10,000 ppm range and <2% of the reading in the 10,000–100,000 ppm range from −20 °C to 50 °C. The sensor has good humidity resistance and can stabilize readings immediately after power-up with a repeatability error <0.6%. In addition, current ultra-compact CO_2_ sensors still have limitations for portable exhaled breath monitoring. Although the response time of ultra-compact CO_2_ sensors has been greatly reduced, it remains insufficient to depict the full waveform information of exhaled breath CO_2_. Increasing the modulation depth of the MEMS emitter, decreasing the response time constant of the thermopile infrared detector, adjusting the vents in the optical chamber, and changing the digital filtering algorithms can improve the response time of the sensor, and we will investigate this issue in subsequent works to completely sample the exhaled CO_2_ waveform.
